# Bereavement due to child loss, divorce, and depressive mood in older age across European welfare regimes

**DOI:** 10.1016/j.ssmph.2024.101721

**Published:** 2024-10-24

**Authors:** Enrico Ripamonti, Mikael Rostila, Jan Saarela

**Affiliations:** a Department of Economics and Management, University of Brescia, Brescia, Italy; b Milan Center for Neuroscience, University of Milan-Bicocca, Milan, Italy; c Department of Public Health Sciences, Stockholm University, Stockholm, Sweden; d Centre for Health Equity Studies (CHESS), Stockholm University/Karolinska Institutet, Stockholm, Sweden; e Aging Research Center, Karolinska Institutet/Stockholm University, Stockholm, Sweden; f Demography Unit, Åbo Akademi University, Vaasa, Finland

**Keywords:** Bereavement, Divorce, Gender, Aging, Depressive symptoms, Welfare regimes

## Abstract

While bereavement, particularly the loss of a child, is a well-known risk factor for mental health in the short term, its long-term consequences on depressive mood in old age and across different welfare regimes have been investigated less. This study focused on the combined role of child loss and divorce on depressive symptoms, measured using the EURO-D scale in Central, Nordic, Southern, and Eastern European countries. We used data from the European SHARE project, covering 22,959 participants aged 50+ over a 16-year period. Using OLS regressions, we found that, compared to no child loss and no divorce, the association between depressive symptoms and child loss was significant (β = 0.22, 95% C.I. = [0.13, 0.30]), among both women and men. The absolute increase was even stronger when the mutual effect of child loss and divorce was considered (β = 0.34, 95% C.I. = [0.18, 0.48]). Employing Generalized Estimating Equations, we found that depressive symptoms related to divorce did not increase over time, regardless of past bereavement. Compared with people in the Nordic countries, those living in Southern Europe experienced more depressive symptoms related to child loss and no divorce, but fewer depressive symptoms related to the combined effect of child loss and divorce. In sum, our findings indicate that bereavement due to child loss may lead to more depressive symptoms among both women and men in old age, especially in combination with divorce. In the latter case, we posit that participants living in Southern European countries may be protected by higher levels of social support through family ties or informal social networks.

## Highlights

• Child loss is linked to increased depression in the elderly.
• Combined effects of child loss and divorce heighten depressive symptoms.
• Divorce-related depression does not worsen over time.
• Northern Europeans face less depression from child loss alone.
• Strong social ties in Southern Europe mitigate effects of loss and divorce.

## 1 Introduction

The death of a child is one of the most devastating and traumatic events in life (Rubin, 1993). Child loss may affect the risk of parental divorce, as well as couples’ likelihood of giving birth to another child (Finnäs et al., 2018). An increased risk of mortality following child loss has also been observed (Donnelly et al., 2020; Li et al., 2003; Rostila et al., 2012; Song et al., 2019). Stressful life events, like child loss, have been shown to co-occur with the onset of depressive episodes (Kendler et al., 1999), distress, and anxiety (Gold et al., 2016; Kreicbergs et al., 2004; Vance et al., 1995). Child loss is also associated with increased use of psychotropic drugs, such as antidepressants and anxiolytics, especially during the first year after bereavement (Rostila et al., 2018).

The loss of a child has significant consequences on parental mental health in the first few years after the event (Stroebe et al., 2007; Wall-Wieler et al., 2018). However, the *sequelae* of child loss on mental health across the life course, and particularly the presence of depressive symptoms in older age, is still a relatively unexplored issue. Studying how past bereavement due to child loss is associated with depressive symptoms in old age could be crucial for mental health prevention and for identifying strategies that may buffer detrimental effects.

Research has highlighted the characteristics of depressive mood in late life (for review see Alexopoulos, 2019; Blazer, 2000). Fiske et al. (2009) and Naismith et al. (2012) found that grief is frequently triggered in old age, which may be due to a general decline in mental health or to increased psychological vulnerability. Additionally, Alpass and Neville (2003) reported that older, retired individuals may often be more exposed to the harmful effects of increased social isolation. Dannefer (2003) and Ferraro and Shippee (2009) highlighted that other negative life events can further deteriorate individuals’ mental health.

This study takes a life course perspective on depressive symptoms in older age (Coman & Ataullahjan, 2010; Kessler et al., 2010), in which these are regarded as arising due to a complex interplay of events and exposures over one's lifetime. In particular, we focused on the possible role of child loss and divorce occurrence. The relationship between these two events is a debated issue in the literature. While some studies have indicated that child loss may increase cohesion and solidarity within the couple, thus reducing the risk of divorce (Anderson et al., 2005; Lyngstad, 2013), large-scale empirical research conducted in Sweden has found a link between child loss and a higher probability of divorce (Van den Berg et al., 2017). Another study, conducted in Finland and focusing on couples of childbearing age, found only a modest association (Finnäs et al., 2018). It has also been found that bereavement increases the risk of marital problems and divorce (e.g., Li et al., 2005). The role of divorce as an added burden that may lead to increased depressive symptoms is particularly worth investigating. Divorce has been described as a potentially stressful event in people's lives, even though there is high variability in the reactions to couple separation (Amato, 2000). The potential liberating effect of divorce has also been highlighted (e.g., Albeck & Kaydar, 2002; Kalmijn & van Groenou, 2005). So, here we aim to investigate whether the joint effect of bereavement and divorce may lead to worse depressive mood.

Few studies have explored cross-national differences in the mental health outcomes associated with bereavement, despite significant variations in welfare services and informal social support systems across countries (Rostila, 2015). Notably, welfare regimes (Esping-Andersen, 1990; Ferrera, 1996) such as those in the Nordic countries offer universal and generous support systems that may mitigate the adverse effects of bereavement and divorce. These systems include benefits like sick leave and compensation following a child's death, provided through robust healthcare systems. However, even within this regime type, the level of public health support can vary significantly (Lyttkens et al., 2016). At the same time, liberal regimes typified by countries like the United Kingdom and Ireland make bereavement or divorce a problem that the individual is left to tackle on his/her own, or with support from immediate family. This individualistic approach may significantly affect the grieving process. By contrast, Southern European countries may provide stronger informal social support because of their family-oriented nature. Moreover, parents from these countries may rely more on extended family, friends, informal networks, and communities for bereaved/divorced in the absence of developed welfare services. Countries with minimal social security systems, such as those in Eastern Europe (Eikemo et al., 2008), present a starkly different scenario. The absence of substantial social security can limit individuals' opportunities and time to grieve effectively, compounded by economic pressures such as the risk of income loss and unemployment. Finally, Bismarkian welfare regimes (Central European countries) are characterized by a social welfare founded upon the strong role of the employer as well as work-related benefits. The insurance obligation is triggered automatically with the start of an income-producing occupation. For those who lose their insurance in these countries there is a welfare safety net, albeit less robust than the type found in Scandinavian countries. At the same time, similar to Mediterranean Europe, these countries are characterized by strong, conservative family bonds. We hypothesize that, given the absence of a strong national healthcare system comparable to those in the Nordic countries, people living in Central European countries could be less protected than those in the Nordic countries in terms of the putative association between bereavement and depression. Given these variations, a comparative analysis of welfare regimes could be particularly informative, clarifying how differences in social security may affect the possibility to grieve and highlighting the contrasting experiences across welfare models.

This study aims to assess the association between exposure to past child bereavement and the risk of depressive symptoms across welfare state regimes in a large, multi-population sample of individuals in the transition phase (50–60 years old) or in older age (60+) (WHO, 2023). Furthermore, we study the correlation between child loss and depressive symptoms in transition/old age among divorced individuals, and explore possible gender differences.

## 2 Methods

### 2.1 Database

We used the Survey of Health, Ageing, and Retirement in Europe (SHARE) (Börsch-Supan et al., 2013), a household-based prospective multi-population panel database of microdata, with data collection spanning from Wave 1 in 2004/05 to Wave 8 in 2019/20 (see the Supplementary Information). For the purposes of the present investigation, following the typology of Ferrera (1996) we considered data from two participating Nordic European countries (Denmark, Sweden), six Central European countries (Austria, Belgium, France, Germany, the Netherlands, Switzerland), and three Southern European countries (Greece, Italy, Spain). These countries participated in all SHARE assessments. Data from two Eastern European countries (the Czech Republic and Poland) were also added. These countries have participated in SHARE since Wave 2, and were added in order to investigate putative differences across welfare regimes (Eikemo et al., 2008).

### 2.2 Measures

*Assessment of bereavement and exposure definition.* The presence of past bereavement due to child loss was retrospectively ascertained through the SHARELIFE (Wave 3) questionnaire; see E-Fig. 1. In this questionnaire, for each of their children, participants declare whether he/she is still alive, and if not, the year in which the death occurred. The exposure variable was created based on past bereavement due to child loss and divorce, respectively; i.e., in terms of a dichotomized indicator (no or yes). This variable has four categories: (i) no child loss, no divorce; (ii) child loss, no divorce; (iii) no child loss, divorce; and (iv) child loss, divorce. We also examined the possible role of child loss and divorce in regard to depressive symptoms in a number of subgroups. First, we considered the age of the child when he/she died, using the categories (i) no child loss; (ii) child died at age 0–6; (iii) child died at age 7–17; and (iv) child died at age 18+. We used this classification in order to distinguish between early childhood (0–6), childhood and adolescence (7-17), and late adolescence, early adulthood, adulthood (18+). Second, we considered the time since the child's death. This variable was categorized as (i) no child loss; (ii) child died fewer than six years ago; (iii) child died six to ten years ago; or (iv) child died at least ten years ago. These categories allowed us to distinguish between a recency effect and older events.

*Assessment of depressive symptoms and outcome definition.* The study outcome is depressive symptoms as measured using the EURO-D scale (Prince et al., 1999). This scale, developed to harmonize the assessment of late-life depression across European countries, is made up of the items depression, pessimism, suicidality, guilt, sleep, interest, irritability, appetite, fatigue, concentration, enjoyment, and tearfulness. Each item is scored 0 (symptom not present) or 1 (symptom present). The EURO-D scores range from 0 to 12, and can be treated as continuous scores or dichotomized following standard criteria, which usually implies that a EURO-D score of 4 or higher indicates the presence of depressive symptoms. To mitigate the effect of skewness, the EURO-D scores were square-root transformed.

*Covariates and other variables*. These included country, birth cohort (recategorized in 6 levels), age, gender, educational level (years of education, recategorized in quintiles), marital status (classified in the SHARE questionnaires as “married, living with spouse”; “registered partnership”; “married not living with spouse”; “divorced”; “never married”; “widowed” – recategorized as “living with partner”; and “not living with partner”), presence of chronic diseases (classified as 0, 1, 2+), smoking (ever smoked daily: “yes” or “no”), and drinking (more than 2 glasses of alcohol almost every day: “yes” or “no”).

### 2.3 Statistical methods

*Descriptive analysis.* Baseline characteristics of the sample stratified by gender were reported in terms of descriptive indicators like median, inter-quartile range, mean, and standard deviation. Missing values in the covariates were replaced with a multiple imputation procedure (five plausible values) already available in the online SHARE database. No imputation was done for the exposure variables, and imputed values for these are not available for the SHARELIFE dataset.

*Multivariable analysis*. Considering continuous EURO-D scores (square-root transformed), the association between bereavement due to past child loss (independent variable) and the presence of depressive symptoms was evaluated using OLS regression with robust standard error estimation (clustering participants by country). Analyses were first run unadjusted and were then adjusted by gender, age, educational level, birth cohort, marital status, number of chronic diseases, drinking, and smoking. Models were re-estimated by inserting an interaction between exposure and welfare regime. Then, we analyzed the trajectory of depressive symptoms over time, considering repeated measures for each participant across six SHARE follow-up assessments (Waves 2, 4, 5, 6, 7, and 8). For this purpose, we used Generalized Estimating Equations (GEEs). Age was set as the underlying time scale and was inserted as an interaction with the exposure and with the welfare regime (exposure × age × welfare regime), in order to assess possible changes over time across welfare regimes.

*Supplementary analyses*. We ran supplementary analyses, stratifying the sample by gender. Additionally, we stratified the sample by participants aged 50–60 vs. those aged 60+ at the first SHARE assessment. The World Health Organization defines older age as 60+ and refers to the 50s as a “transition” age (WHO, 2023). While this cut point is not univocal (see, for instance, European Commission, 2021), we followed the WHO's distinction in the present study. We also ran a sensitivity analysis considering dichotomizing EURO-D scores using the standard procedure and estimating logistic regression models. The results of the sensitivity analyses are described in the Supplementary Information.

*Reporting.* The study was conducted in accordance with the Strengthening the reporting of observational studies in epidemiology (STROBE) guidelines (Von Elm et al., 2007). A *p*-value of p < 0.05 was used to indicate statistical significance for each test. Analyses were performed using the SAS 9.4 software.

## 3 Results

### 3.1 Descriptive analysis

**Fig. 1:**
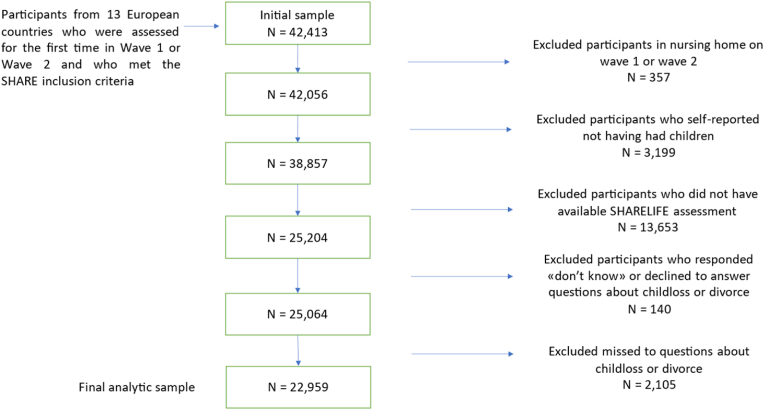
Flowchart illustrating the creation of the analytical sample with inclusion/exclusion criteria.

From the selected countries, we initially selected 42,413 participants in Wave 1 or 2 (baseline evaluation). We excluded those who lived in a nursing home (n = 357), which led to a sample of 42,056 participants. The rationale for this exclusion criterion is that, compared to community-dwelling individuals, institutionalized elderly people may have other main drivers for depressive symptoms such as severe disease and cognitive impairment (Barca et al., 2009). We then eliminated participants who reported never having had children (3,199) and those who did not have an available SHARELIFE assessment in Wave 3 (13,653). Finally, we removed participants who had answered “don't know” or had declined to answer (n = 140) or missed (n = 2105) the questions about child loss and divorce. This led to a final analytic sample of 22,959 participants (see Fig. 1). Of these participants, 9758 (42.5%) were from Central European countries, 3439 (15.0%) were from Nordic European countries, 6588 (28.7%) were from Southern European countries, and 3174 (13.8%) were from Eastern European countries (see Table 1 and E-Table 1).

**Table 1:** Descriptive statistics of the exposure variables for participants who were assessed at baseline (Wave 1 or 2), all countries and by welfare regime.

Variable	Levels	All countries	Central European welfare	Nordic European welfare	Southern European welfare	Eastern European welfare
		N = 22,959	N = 9758	N = 3439	N = 6588	N = 3174
*Child loss combined with divorce occurrence*	No child loss, no divorce	18,758(81.7%)	7643(78.3%)	2358(73.8%)	5965(90.5%)	2612(82.3%)
	Child loss, no divorce	1428(6.2%)	642(6.6%)	138(4.0%)	394(5.9%)	254(8.0%)
	No child loss, divorce	2590(11.3%)	1365(14.0%)	717(20.8)	220(3.3%)	288(9.1%)
	Child loss, divorce	183(0.8%)	108(1.1%)	46(1.3)	9(0.1%)	20(0.6%)

*Child loss according to age*	No child loss	21,348(92.9%)	9008(92.3%)	3255(94.6%)	6185(93.9%)	2900(91.4%)
	Child loss, decease occurred at age 0-6	850(3.7%)	382(3.9%)	77(2.2%)	242(3.7%)	149(4.7%)
	Child loss, decease occurred at age 7-17	156(0.7%)	66(0.7%)	30(0.8%)	36(0.5%)	24(0.8%)
	Child loss, decease occurred at age 18+	605(2.6%)	302(3.1%)	77(2.4%)	125(1.9%)	101(3.2%)

*Time since child death*	No child loss	21,348(92.9%)	9008(92.3%)	3255(94.6%)	6185(93.9%)	2900(91.4%)
	Child loss, decease occurred less than 6 years from SHARELIFE	515(2.2%)	245(2.5%)	59(1.7%)	124(1.9%)	87(2.7%)
	Child loss, decease occurred 6–10 years from SHARELIFE	126(0.5%)	52(0.5%)	13(0.4%)	37(0.6%)	24(0.8%)
	Child loss, decease occurred 10+ years from SHARELIFE	970(4.2%)	453(4.6%)	112(3.3%)	242(3.7%)	163(5.1%)

*Time since divorce*	No child loss or no divorce	22,776(99.2%)	9650(98.9%)	3393(98.7%)	6579(99.9%)	3154(99.4%)
	Divorce occurred before child loss	99(0.4%)	61(0.6%)	21(0.6%)	3(0.1%)	14(0.4%)
	Divorce occurred after child loss	77(0.3%)	46(0.5%)	21(0.6%)	5(0.1%)	5(0.2%)
	Divorce and child loss occurred the same year	7(0.03%)	1(0.01%)	4(0.1%)	1(0.02%)	1(0.03%)

There were 9931 male participants (43.3%), and 13,028 female participants (56.7%); see E-Table 2. The median age at baseline was 62 years (Q1 = 55; Q3 = 70). Most participants lived with a partner (n = 17,873, 77.8%). The median number of children was 2, and the median number of years of education was 11. The majority of participants were retired (n = 10,740, 46.8%), while a lower proportion were employed or self-employed (n = 7,081, 30.8%). With the standard cut-off of ≥ 4 for the EURO-D scale, 5731 (25.0%) participants were above the cut-off (among whom 1543 were men and 4188 women). The distribution of EURO-D scores by welfare regime and gender is shown in E-Fig. 2.

As to the exposure variables (Table 1), 18,758 participants (81.7%) had not experienced child loss or divorce, 1428 (6.2%) had experienced child loss but not divorce, 2590 (11.3%) had not experienced child loss but divorce, and 183 (0.8%) had experienced both child loss and divorce. For 850 participants (3.7%) the decease had occurred when the child was aged 0–6, for 156 (0.7%) when the child was aged 7–17, and for 605 (2.6%) when the child was aged 18+. For 515 participants (2.2%) the decease had occurred fewer than six years before the SHARELIFE interview, for 126 (0.5%) 6–10 years before the interview, and for 970 (4.2%) ten or more years before the interview. For 99 participants (0.4%) divorce had occurred before child loss, for 77 (0.3%) divorce had occurred after child loss, and for 7 the two events had occurred in the same year.

### 3.2 Multivariable analysis

We first estimated OLS regression at baseline (Wave 1 or 2), considering child loss combined with divorce occurrence as exposure. Compared to no experience of child loss and divorce, having experienced child loss (only) was found to increase the average EURO-D (on the square root scale) score by 0.22 units (β = 0.22 95% C.I.:[0.13, 0.30]; see Table 2). The unstandardized β refers to absolute sizes in estimated effects. This corresponds to a standardized effect size of 0.06 standard deviations ($\beta_{ST}=0.06$). Having experienced both child loss and divorce had an even stronger association with depressive symptoms, or a 0.34 unit absolute increase in the average EURO-D score (β = 0.34[0.18, 0.48], $\beta_{ST}=0.03$).

**Table 2:** Estimates of effect of exposure to child loss/divorce on presence of depressive symptoms (square-root transformed EURO-D scores) at baseline (Wave 1 or 2).

Unadjusted		Exposure∗Welfare regime			

Exposure	All countries	Central	Nordic	Southern	Eastern

No child loss, no divorce	Ref.	Ref.	Ref.	Ref.	Ref.
Child loss, no divorce	0.31[0.21, 0.39]∗	0.01[-0.12, 0.14]	−0.01[-0.14, 0.12]	0.22[-0.0009, 0.45]	0.18[-0.02, 0.39]
No child loss, divorce	0.03[-0.05, 0.12]	0.08[-0.01, 0.17]	−0.08[-0.17, 0.01]	−0.08[-0.18, 0.01]	−0.16[-0.24, −0.09]∗
Child loss, divorce	0.31[0.16, 0.46]∗	0.16[-0.02, 0.35]	−0.16[-0.35, 0.02]	0.14[-0.04, 0.33]	0.09[-0.09, 0.27]


Adjusted		Exposure∗Welfare regime			

Exposure	All countries	Central	Nordic	Southern	Eastern

No child loss, no divorce	Ref.	Ref.	Ref.	Ref.	Ref.
Child loss, no divorce	0.22[0.13, 0.30]∗	0.007[-0.20, 0.22]	−0.007[-0.22, 0.20]	0.20[0.03, 0.37]∗	0.15[0.009, 0.29]∗
No child loss, divorce	−0.003[-0.09, 0.08]	0.05[-0.06, 0.17]	−0.05[-0.17, 0.06]	0.25[0.11, 0.39]∗	−0.14[-0.26, −0.03]∗
Child loss, divorce	0.34[0.18, 0.48]∗	0.02[-0.29, 0.33]	−0.02[-0.33, 0.29]	−0.90[-1.24, −0.57]∗	−0.12[-0.42, 0.18]

Beta estimates and confidence intervals (in brackets) are shown. Estimates are from OLS regression with robust standard error estimation (unadjusted, above; adjusted, below). These have been obtained for all countries and when using an interaction with welfare regime. In the column “All countries” there is no interaction with welfare regime. In the columns “Exposure∗Welfare regime”, “Nordic” welfare regime has been taken as a reference level and its own effect has been obtained switching the reference to “Central” welfare regime. The reference level for the Exposure has been kept fixed at level “No child loss, no divorce”. In the panel “Adjusted”, adjustment is by age, birth cohort, gender, education, marital status, smoking, drinking, chronic diseases. Observations were clustered by country for robust standard error estimation.

Interaction analysis (Type-III test, F(9,12) = 144.25, *p* < 0.0001) indicated that the effect of the exposure varies across welfare regimes. We did not detect differences between the Northern and Central welfare regimes, however (Table 2, E-Fig. 3). Interestingly, compared with participants living in Nordic European countries, those living in Southern European countries who had experienced child loss but no divorce showed relatively more depressive symptoms (β = 0.20[0.03, 0.37], $\beta_{ST}=0.03$). The same was true of participants who had experienced no child loss but divorce (β = 0.25[0.11, 0.39], $\beta_{ST}=0.03$). However, the direction of this effect was the reverse for participants who had experienced both child loss and divorce (β = −0.90[−1.24, −0.57], $\beta_{ST}=-0.01$). As to participants living in Eastern welfare regimes, compared to the Nordic welfare regime we found more depressive symptoms among those who had experienced child loss but no divorce (β = 0.15[0.009, 0.29], $\beta_{ST}=$ 0.03) and fewer depressive symptoms for those who had experienced no child loss but divorce (β = −0.14[−0.26, −0.03], $\beta_{ST}=$-0.03).

We then estimated GEEs to evaluate change over time. While in the case of no child loss and no divorce we found a marginal annual increase in depressive symptoms, quantified as about 0.0052 points (i.e., β = 0.0052[0.004, 0.0065]) of the square root of the EURO-D score, the interaction effects revealed significant differences across exposure groups (Table 3, E-Fig. 4). The Type-III test for the exposure × age interaction was significant (F(3,61139) = 9.27, *p* < 0.0001). In the case of divorce but no child loss, the observed age-related increase in depressive symptoms compared to the reference group was almost zero (β = −0.005[−0.007, −0.002]), implying that depressive symptoms were quite stable over time for these participants. A more pronounced effect was observed in the group of participants who had experienced both divorce and child loss, in which the interaction term (β = −0.012[−0.019, −0.005]) suggested not only an offset but a reversal of the longitudinal effect observed in the reference group. Consequently, for these participants, each additional year is associated with a marginal decrease by about 0.007 units in the square root of the EURO-D score, highlighting a slight reduction in depressive symptoms over time in this group compared to the reference group.

**Table 3:** Estimates of effect of exposure to child loss/divorce on presence of depressive symptoms (square-root transformed EURO-D scores) at baseline (Wave 1 or 2) in interaction with age (longitudinal effects) and welfare regime.

Unadjusted		Exposure∗Age∗Welfare regime			

Exposure∗Age	All countries	Central	Nordic	Southern	Eastern

No child loss, no divorce	Ref.	0.0015[-0.007, 0.004]	−0.0015[-0.004, 0.0007]	0.007[0.004, 0.009]∗	0.004[0.0008, 0.006]∗
Child loss, no divorce	0.0008[-0.002, 0.004]	−0.003[-0.01, 0.006]	0.003[-0.006, 0.01]	0.00003[-0.009, 0.009]	−0.006[-0.02, 0.005]
No child loss, divorce	−0.007[-0.009, −0.005]∗	−0.0004[-0.005, 0.004]	0.0004[-0.004, 0.005]	0.004[-0.005, 0.01]	0.0003[-0.008, 0.009]
Child loss, divorce	−0.014[-0.02, −0.006]∗	−0.001[-0.02, 0.01]	0.001[-0.01, 0.02]	0.04[0.02, 0.06]∗	0.01[-0.02, 0.04]


Adjusted		Exposure∗Age∗Welfare regime			

Exposure∗Age	All countries	Central	Nordic	Southern	Eastern

No child loss, no divorce	Ref.	0.002[-0.0003, 0.004]	−0.0019[-0.004, 0.0003]	0.007[0.004, 0.009]∗	0.008[0.005, 0.011]∗
Child loss, no divorce	−0.001[-0.004, 0.002]	−0.004[-0.01, 0.005]	0.004[-0.005, 0.01]	−0.001[-0.01, 0.008]	−0.001[-0.01, 0.009]
No child loss, divorce	−0.005[-0.007, −0.002]∗	−0.0007[-0.005, 0.004]	0.0007[-0.004, 0.005]	0.002[-0.008, 0.011]	0.007[-0.001, 0.01]
Child loss, divorce	−0.012[-0.019, −0.005]∗	0.0004[-0.01, 0.016]	−0.0004[-0.01, 0.01]	0.04[0.004, 0.08]∗	0.007[-0.02, 0.03]

Beta estimates and confidence intervals (in brackets) are shown. Estimates are from Generalized Estimating Equations (GEEs): longitudinal effects (interaction with the time scale). These have been obtained for all countries and repeated, using an interaction with welfare regime (unadjusted, above; adjusted, below). In the column “All countries” there is no interaction with welfare regime. In the column “Exposure∗Age”, the reference level for Exposure is “No child loss, no divorce”. In the columns “Exposure∗Age∗Welfare regime”, “Nordic” welfare regime has been taken as a reference level and its own effect has been obtained by switching the reference to “Central” welfare regime.

We repeated the analysis, adding the interaction term with the welfare regime. The Type-III test for the three-way interaction was significant (F(12,850) = 4.98, *p* < 0.0001). In the case of child loss or divorce, we did not detect significant differences among the Central, Nordic, and Eastern welfare regimes. However, for the group of participants who had experienced both child loss and divorce, we found a significant effect (β = 0.04[0.004, 0.08]) among those from Southern European countries compared to those from Nordic countries. The coefficient 0.04 can be interpreted as an additional increase in the outcome for each unit increase in age among those at the specified level of exposure (i.e., child loss and divorce) and living in the Southern welfare regime compared to those living in the Nordic one.

We considered the other categories of exposure described in the Methods section, and estimated OLS regression at baseline (E-Table 3). Compared to no experience of child loss, a child's death at age 0–6 years and age 18+, respectively, had positive associations with depressive symptoms (β = 0.26[0.14, 0.38], $\beta_{ST}$ = 0.06 and β = 0.29[0.23, 0.37], $\beta_{ST}$ = 0.05). Child deaths that occurred 6–10 years and 10+ years, respectively, since SHARELIFE assessment were also positively associated with depressive symptoms (β = 0.35[0.14, 0.55], $\beta_{ST}$ = 0.03 and β = 0.27[0.16, 0.39], $\beta_{ST}$ = 0.06). Divorce before child loss and divorce after child loss were both associated with depressive symptoms (β = 0.41[0.22, 0.66], $\beta_{ST}$ = 0.03 and β = 0.37[0.02, 0.73], $\beta_{ST}$ = 0.02). In E-Table 3 we also show the results of the interaction exposure × welfare regimes. Again, significant differences emerged only when the Nordic and the Southern welfare regimes were compared.

### 3.3 Analysis stratified by gender

We repeated the main analysis at baseline, stratifying by gender (E-Table 4). The association between experience of child loss and depressive symptoms was positive and significant for both females (β = 0.11[0.01, 0.22], $\beta_{ST}$ = 0.03) and males (β = 0.22[0.08, 0.35], $\beta_{ST}$ = 0.06). The same was found for the experience of both child loss and divorce (female: β = 0.35[0.19, 0.49], $\beta_{ST}$ = 0.04 male: β = 0.36[0.07, 0.64], $\beta_{ST}$ = 0.03). No association was found for the experience of divorce only, among either females or males. The interaction analysis (exposure × welfare regime) for females (Type-III test, F(9,12) = 781, *p* < 0.0001) indicated that, compared to women in the Nordic countries, those in Southern Europe showed more depressive symptoms related to child loss only (β = 0.14[0.007, 0.27], $\beta_{ST}$ = 0.02) and to divorce only (β = 0.30[0.12, 0.48], $\beta_{ST}$ = 0.03), and fewer depressive symptoms related to the experience of both child loss and divorce (β = −1.16[−1.39, −0.92], $\beta_{ST}$ = −0.02).

### 3.4 Analysis stratified by age group

We also stratified the analysis by age group. For those aged 50–60 years at first assessment (E-Table 5) the experience of child loss was associated with depressive symptoms (β = 0.30[0.19, 0.41], $\beta_{ST}$ = 0.06), as was the experience of both child loss and divorce (β = 0.44[0.11, 0.77], $\beta_{ST}$ = 0.04). Similar results were obtained for those who were aged 60+ at first assessment (β = 0.18[0.09, 0.27], $\beta_{ST}$ = 0.06 and β = 0.27[0.12, 0.42], $\beta_{ST}$ = 0.03 in E-Table 5). For this age class, it is worth reporting results relative to the interaction exposure × welfare regime (Type-III test, F(9,12) = 411, *p* < 0.0001). Participants in Southern Europe showed more depressive symptoms related to the experience of divorce than did those in the Nordic countries (β = 0.46[0.36, 0.57], $\beta_{ST}$ 0.04), but fewer depressive symptoms related to the experience of both child loss and divorce (β = −0.79[−1.15, −0.44], $\beta_{ST}$ = −0.02). Other findings and sensitivity analyses are reported in E-Table 6 and E-Table 7 in the Supplementary Information.

## 4 Discussion

As Europe's population ages, it is expected that the prevalence and societal impact of common conditions like depression will gradually rise (Naismith et al., 2012). The presence of depressive symptoms in old age is often a reaction to functional impairment and declining health (Alexopoulos, 2005, 2019). However, the effect of life events and their negative impact on chronic stress may also contribute to the emergence of depressive mood (Marin et al., 2011). This study focused on the combination of two negative life events, child loss and divorce, and their association with depressive symptoms in old age. This has been framed at a macro level considering the role of differing welfare regimes in moderating the association.

Our findings reveal that past bereavement due to child loss may independently predict depressive symptoms in older age. However, we found that the combination of two of the most stressful life events an individual can experience – child loss and divorce – is particularly detrimental to the risk of depression in people aged 50+. The stage model of grief (Kübler-Ross & Kessler, 2014) conceives of depressive mood as a transitory and identifiable state following child loss. The same is true of the disorganization-reorganization model (Temes, 1992). By contrast, our results indicate that depressive symptoms might not be temporary or restricted to a certain stage after child loss and/or divorce but rather that the consequences of such stressful life events are long-term (Moor & de Graaf, 2016), especially if they co-occur. This may indicate a pattern of chronic dysfunction, distress, unresolved or prolonged/delayed grief (Field, 2006), immobilization, pain, coping efforts, and accommodation among these individuals (Bonanno, 2004; van der Houwen et al., 2010). Along these lines, it is known that grief does not always follow an identifiable period and can extend over several years (Cha & Thomas, 2023; Rubin, 1999). Thus, the data are in agreement with a continuous accommodation (Walsh & McGoldrick, 2013) and task-based approach to grief and loss (Worden, 2018), which conceives of bereavement as a process rather than a series of discrete stages. Moreover, previous experiences/negative life events can trigger grief and thereby depressive symptoms at specific time points such as anniversaries (Rostila et al., 2015).

In regard to the exposure to both past bereavement and divorce, it is worth noting that as the total sample size for this group (n = 183) was low, these results need further investigation. Nevertheless, these individuals deserve careful monitoring from a public health perspective. We found that the combination of child loss and divorce led to more depressive symptoms (in terms of absolute increase) than child loss and no divorce. One might ask whether the timing of the divorce affects the association; we targeted this specific issue in the analyses, but found that depressive symptoms were present in participants regardless of the timing of the divorce. For those who experience divorce before child loss we might hypothesize that these people may be particularly fragile, and that when child loss happens they thus may struggle to regain their balance and reconfigure their relationships and interactional processes (Schiffman, 2019). This group could constitute a selection of individuals who have experienced complicated grief or a difficult grief process. For those who experience divorce after child loss, the child loss itself, with the challenges it entails, may be the reason for the divorce.

The longitudinal analysis indicates that in our sample, among individuals who experienced divorce (with or without child loss), depressive symptoms were stable or progressively slightly decreased over time. We might hypothesize that this is a consequence of the activation of resilience mechanisms (Maccallum et al., 2015; Mancini et al., 2012) in response to facing stressful and adverse circumstances, or that it may simply be due to the transition into a different life phase, in which there is a different modulation and interplay among past life events, life scars, and mental health.

The analysis stratified by gender revealed that bereavement can be an extensive, prolonged condition for both women and men. The literature has already documented that, among women, depressive symptoms in old age occur as a consequence of bereavement, possibly complicated grief (Mellencamp, 2023; Shear, 2015; Zetumer et al., 2015), independent of divorce. It is well known that fathers and mothers grieve differently (Alam et al., 2012), and that a mother's attachment is more intimate and intense (Schiffman, 2019). This, again, may underline the protective role of family and informal networks. A study conducted in Taiwan found that depressive symptoms affected mothers but not fathers (Lee et al., 2014). Contrary to this assumption, our results indicate that in terms of depressive symptoms there are no relevant gender differences in older age. Indeed, the present data indicate that bereaved fathers also constitute a group of individuals who are particularly at risk of depressive mood (McNeil et al., 2021; Park & Kim, 2024), and should be monitored over time.

This study also explored the role of different European welfare regimes in moderating the relationship between bereavement, divorce, and depressive symptoms. In general, our findings showed no significant differences in depressive symptoms between Nordic and Central European countries in both the cross-sectional and longitudinal analyses across different levels of the exposure (bereavement and/or divorce). However, participants from Southern European countries who had experienced child loss showed higher levels of depressive symptoms compared to bereaved individuals living in Nordic and Central European countries. Participants from Eastern European countries who had experienced child loss also reported higher depressive symptoms than those living in Nordic (and Central) European countries. A different picture was observed in the case of bereavement and divorce, in which we found a protective effect of living in Southern European countries.

We might assume that a strong welfare system may, at least in part, compensate for the effects of negative events like bereavement and divorce. This was confirmed by our results among individuals experiencing either child loss or divorce and living in the Nordic countries, who were relatively protected compared to those living in Southern or Eastern European welfare regimes. This suggests that the extensive and universal welfare support in the Nordic countries might better buffer the adverse effects of child bereavement. This picture was substantially confirmed in Central European countries, which showed levels similar to those obtained in the Nordic countries. Central European countries like Germany do not operate under the same universal welfare regime as the Nordic countries, but rather a Bismarkian type of regime founded on work-related insurance and basic safety services for all citizens. The levels of public expenses are comparable between the two models (OECD, 2023). Our results seem to indicate that living in either of these welfare regimes, regardless of the specific organizational model, leads to the provision of a similar level of protection for individuals who have experienced child loss. Both the Nordic and Central European welfare regimes may also provide better protection than the Southern and Eastern European ones during illness and poor health (e.g., in terms of sickness benefits).

Interestingly, in the case of a double burden – i.e., for those individuals experiencing both bereavement and divorce – living in Southern European countries was associated with fewer depressive symptoms at baseline. This result was significant in the subsample of female participants as well. For this specific exposure category, this may indicate a protective effect of a welfare regime that relies on strong family connections, informal networks, horizontal subsidiarity, and the actions of charities and non-governmental organizations. Our results seem to indicate that, for individuals who experienced both these negative events, social support and informal ties may be relatively more important than a structured system of welfare services. A cautionary note regarding interpreting this result, however: while the protective effect of the Southern welfare regime was highlighted at baseline, longitudinally we found a slight increase in depressive symptoms among these participants.

Results also indicate that living in post-communist Eastern welfare regimes may lead to more depressive symptoms in the case of child loss. This is in line with the expectation that absent or scarce social security impacts the possibility for elaborate grief. Surprisingly, participants from Eastern countries who had experienced divorce with no child loss showed fewer depressive symptoms than those living in other welfare regimes: a result that deserves further investigation.

The type of healthcare system in place is a significant contextual-level determinant of health (CSDH, 2008). Additionally, the roles of states, regions, and municipalities within each welfare regime is important (Pronk et al., 2021). Healthcare expenses can serve as a proxy for public investment in health. Using this proxy, the classification of states in this study would closely align with that of welfare regimes (OECD, 2023). A different picture would be obtained if one considered the reference model of public health. Meanwhile, we can classify a Bismark model (social security system) used by both Central and Eastern European countries, and a Beveridge model (national health system) adopted by Nordic and Southern European countries (Kutzin et al., 2009). Our view is that, given our specific focus, it is more relevant to analyze welfare regimes than healthcare systems. Understanding the impact of bereavement (with or without divorce) requires the consideration of both social services/assistance and the public health system. Moreover, welfare regimes encompass informal networks such as family and friends, which may provide protection. Hence, a four-way classification of welfare regimes is more suitable than a purely two-way classification of healthcare systems.

A strength of the present study is that it used a large representative sample of the European population with cross-national data. Limitations of the SHARE database include the relatively low response rate and relatively high rate of loss to follow-up in the longitudinal assessments, which may have influenced the generalizability of the results. There might also be an issue of internal validity, as some individuals may not have reported their experiences of divorce or child loss, which could affect the accuracy of our findings. The use of self-reported measures, the lack of comprehensive data on other negative life events, and the absence of objective diagnoses of depression throughout the life course may limit the depth of our analysis. Loneliness is another condition that is related to depressive symptoms in older age (Editorial – The Lancet, 2023), even though the direction of the effect is uncertain (Cacioppo et al., 2010; Dahlberg et al., 2014). Unfortunately, we could not study the contribution of loneliness as the SHARE data did not include it in its first four waves. Additionally, the relatively low proportion of participants who had experienced both bereavement and divorce restricted the possibility to robustly address the timing of effects and the role of welfare regimes due to power issues. Omitted mediators/moderators, like the quality of the relationship with the deceased child or divorced partner, co-residence with the deceased child, or information on remarriage and new partners, might also have significantly influenced our study's outcomes. It would also be beneficial for future research to study how other country-level factors such as societal norms, rituals, migration, culture, and religion may contribute to the consequences of past bereavement and divorce on depression in European countries.

In conclusion, our study found that past life events, such as the loss of a child and divorce, contribute to the risk of late-life depressive mood. Living in Nordic European countries, compared to Southern European countries, can lead to fewer depressive symptoms in old age in the case of child loss or divorce. In general terms, the picture in Central European countries is substantially similar to that in Nordic countries. By contrast, living in Southern European countries, characterized by strong familial and community ties, may mitigate the effects of these negative events in cases in which they co-occur. Parents living in Eastern European countries, characterized by low levels of social security, might be particularly at risk in the case of child loss, while the role of divorce in this welfare regime requires further clarification. Policymakers should consider implementing or expanding mental health and social support services (especially in Eastern and Southern Europe) targeted at older adults who have experienced significant life events like child loss and divorce. This could include specialized grief counseling, support groups, and mental health resources tailored to the needs of the elderly. Given the protective role of strong family ties and community networks, especially in Southern European countries, policies should encourage the integration of these sources of social support into formal mental health strategies. This might involve training family members and caregivers in mental health first aid, and fostering community initiatives that enhance social connectivity for older adults.

## CRediT authorship contribution statement

**Enrico Ripamonti:** Writing – review & editing, Writing – original draft, Visualization, Validation, Supervision, Software, Methodology, Investigation, Formal analysis, Data curation, Conceptualization. **Mikael Rostila:** Writing – review & editing, Writing – original draft, Validation, Supervision, Methodology, Investigation, Conceptualization. **Jan Saarela:** Writing – review & editing, Writing – original draft, Validation, Supervision, Methodology, Investigation, Formal analysis, Conceptualization.

## Ethics in publishing

### Reporting standard

The study was conducted in accordance with the STrengthening the Reporting of OBservational studies in Epidemiology guidelines (Von Elm et al., 2007). A p-value of p < 0.05 was adopted as indicating statistical significance for each test. Analyses were performed using the SAS 9.4 software.

### Data access

Data used in this study are publicly available, upon registration, at the SHARE consortium: https://share-eric.eu/

## Sources of funding

Swedish Research Council for Health, Working Life and Welfare (FORTE) grant no. 2022-06397.

## Declaration of competing interest

The authors declare that they have no known competing financial interests or personal relationships that could have appeared to influence the work reported in this paper.

## Data Availability

Data are publicly available https://share-eric.eu/.

## References

[bib1] Alam R., Barrera M., D'Agostino N., Nicholas D.B., Schneiderman G. (2012). Bereavement experiences of mothers and fathers over time after the death of a child due to cancer. Death Studies.

[bib2] Albeck S., Kaydar D. (2002). Divorced mothers: Their network of friends pre-and post-divorce. Journal of Divorce & Remarriage.

[bib3] Alexopoulos G.S. (2005). Depression in the elderly. The Lancet.

[bib4] Alexopoulos G.S. (2019). Mechanisms and treatment of late-life depression. Translational Psychiatry.

[bib5] Alpass F.M., Neville S. (2003). Loneliness, health and depression in older males. Aging & Mental Health.

[bib6] Amato P.R. (2000). The consequences of divorce for adults and children. Journal of Marriage and Family.

[bib7] Anderson M.J., Marwit S.J., Vandenberg B., Chibnall J.T. (2005). Psychological and religious coping strategies of mothers bereaved by the sudden death of a child. Death Studies.

[bib8] Barca M.L., Selbæk G., Laks J., Engedal K. (2009). Factors associated with depression in Norwegian nursing homes. International Journal of Geriatric Psychiatry.

[bib9] Blazer D.G. (2000). Psychiatry and the oldest old. American Journal of Psychiatry.

[bib10] Bonanno G.A. (2004). Loss, trauma, and human resilience: Have we underestimated the human capacity to thrive after extremely aversive events?. American Psychologist.

[bib11] Börsch-Supan A., Brandt M., Hunkler C., Kneip T., Korbmacher J., Malter F., Schaan B., Stuck S., Zuber S. (2013). Data resource profile: The Survey of health, ageing and retirement in Europe (SHARE). International Journal of Epidemiology.

[bib12] Cacioppo J.T., Hawkley L.C., Thisted R.A. (2010). Perceived social isolation makes me sad: 5-year cross-lagged analyses of loneliness and depressive symptomatology in the Chicago health, aging, and social relations study. Psychology and Aging.

[bib13] Cha H., Thomas P.A. (2023). A time of healing: Can social engagement after bereavement reduce trajectories of depression after the death of a child?. The Journals of Gerontology: Series B.

[bib14] Colman I., Ataullahjan A. (2010). Life course perspectives on the epidemiology of depression. Canadian Journal of Psychiatry.

[bib15] Commission on Social Determinants of Health (CSDH (2008). Final report of the commission on social determinants of health.

[bib16] Dahlberg L., Andersson L., McKee K.J., Lennartsson C. (2014). Predictors of loneliness among older women and men in Sweden: A national longitudinal study. Aging & Mental Health.

[bib17] Dannefer D. (2003). Cumulative advantage/disadvantage and the life course: Cross-fertilizing age and social science theory. Journals of Gerontology Series B: Psychological Sciences and Social Sciences.

[bib18] Donnelly R., Umberson D., Hummer R.A., Garcia M.A. (2020). Race, death of a child, and mortality risk among aging parents in the United States. Social Science & Medicine.

[bib19] Editorial – The Lancet (2023). Loneliness as a health issue. The Lancet.

[bib20] Eikemo T.A., Huisman M., Bambra C., Kunst A.E. (2008). Health inequalities according to educational level in different welfare regimes: A comparison of 23 European countries. Sociology of Health & Illness.

[bib21] Esping-Andersen G. (1990).

[bib22] European Commission (2021).

[bib23] Ferraro K.F., Shippee T.P. (2009). Aging and cumulative inequality: How does inequality get under the skin?. The Gerontologist.

[bib24] Ferrera M. (1996). The'Southern model’of welfare in social Europe. Journal of European Social Policy.

[bib25] Field N.P. (2006). Unresolved grief and continuing bonds: An attachment perspective. Death Studies.

[bib26] Finnäs F., Rostila M., Saarela J. (2018). Divorce and parity progression following the death of a child: A register-based study from Finland. Population Studies.

[bib27] Fiske A., Wetherell J.L., Gatz M. (2009). Depression in older adults. Annual Review of Clinical Psychology.

[bib28] Gold K.J., Leon I., Boggs M.E., Sen A. (2016). Depression and posttraumatic stress symptoms after perinatal loss in a population-based sample. Journal of Women's Health.

[bib29] Kalmijn M., Van Groenou M.B. (2005). Differential effects of divorce on social integration. Journal of Social and Personal Relationships.

[bib30] Kendler K.S., Karkowski L.M., Prescott C.A. (1999). Causal relationship between stressful life events and the onset of major depression. American Journal of Psychiatry.

[bib31] Kessler R.C., McLaughlin K.A., Green J.G., Gruber M.J., Sampson N.A., Zaslavsky A.M., Williams D.R. (2010). Childhood adversities and adult psychopathology in the WHO world mental health surveys. British Journal of Psychiatry.

[bib32] Kreicbergs U., Valdimarsdóttir U., Onelöv E., Henter J.-I., Steineck G. (2004). Anxiety and depression in parents 4–9 years after the loss of a child owing to a malignancy: A population-based follow-up. Psychological Medicine.

[bib33] Kübler-Ross E., Kessler D. (2014).

[bib34] Kutzin J., Ibraimova A., Jakab M., O'Dougherty S. (2009). Bismarck meets Beveridge on the silk road: Coordinating funding sources to create a universal health financing system in Kyrgyzstan. Bulletin of the World Health Organization.

[bib35] Lee C., Glei D.A., Weinstein M., Goldman N. (2014). Death of a child and parental wellbeing in old age: Evidence from Taiwan. Social Science & Medicine.

[bib36] Li J., Laursen T.M., Precht D.H., Olsen J., Mortensen P.B. (2005). Hospitalization for mental illness among parents after the death of a child. New England Journal of Medicine.

[bib37] Li J., Precht D.H., Mortensen P.B., Olsen J. (2003). Mortality in parents after death of a child in Denmark: A nationwide follow-up study. The Lancet.

[bib38] Lyngstad T.H. (2013). Bereavement and divorce: Does the death of a child affect parents' marital stability?. Family Science.

[bib39] Lyttkens C.H., Christiansen T., Hâkkinen U., Kaarboe O., Sutton M., Welander A. (2016). The core of the Nordic health care system is not empty. Nordic Journal of Health Economics.

[bib40] Maccallum F., Galatzer-Levy I.R., Bonanno G.A. (2015). Trajectories of depression following spousal and child bereavement: A comparison of the heterogeneity in outcomes. Journal of Psychiatric Research.

[bib41] Mancini A.D., Westphal M., Bonanno G.A. (2012). Loss, trauma, and resilience in adulthood. Annual Review of Gerontology and Geriatrics.

[bib42] Marin M.-F., Lord C., Andrews J., Juster R.-P., Sindi S., Arsenault-Lapierre G., Fiocco A.J., Lupien S.J. (2011). Chronic stress, cognitive functioning and mental health. Neurobiology of Learning and Memory.

[bib43] McNeil M.J., Baker J.N., Snyder I., Rosenberg A.R., Kaye E.C. (2021). Grief and bereavement in fathers after the death of a child: A systematic review. Pediatrics.

[bib44] Mellencamp K.A. (2023). Gender differences in depressive symptoms following child death in later life. The Journals of Gerontology: Series B.

[bib45] Moor N., de Graaf P.M. (2016). Temporary and long-term consequences of bereavement on happiness. Journal of Happiness Studies.

[bib46] Naismith S.L., Norrie L.M., Mowszowski L., Hickie I.B. (2012). The neurobiology of depression in later-life: Clinical, neuropsychological, neuroimaging and pathophysiological features. Progress in Neurobiology.

[bib47] OECD (2023).

[bib48] Park S., Kim J. (2024). The death of an adult child and trajectories of parental depressive symptoms: A gender-based longitudinal analysis. Social Science & Medicine.

[bib49] Prince M.J., Reischies F., Beekman A.T., Fuhrer R., Jonker C., Kivela S.-L., Lawlor B.A., Lobo A., Magnusson H., Fichter M. (1999). Development of the EURO–D scale–a European Union initiative to compare symptoms of depression in 14 European centres. The British Journal of Psychiatry.

[bib50] Pronk N., Kleinman D.V., Goekler S.F., Ochiai E., Blakey C., Brewer K.H. (2021). Promoting health and well-being in healthy people 2030. Journal of Public Health Management and Practice.

[bib51] Rostila M., Mäki N., Martikainen P. (2018). Does the death of a child influence parental use of psychotropic medication? A follow-up register study from Finland. PLoS One.

[bib52] Rostila M., Saarela J., Kawachi I. (2012). Mortality in parents following the death of a child: A nationwide follow-up study from Sweden. Journal of Epidemiology & Community Health.

[bib53] Rostila M., Saarela J., Kawachi I., Hjern A. (2015). Testing the anniversary reaction: Causal effects of bereavement in a nationwide follow-up study from Sweden. European Journal of Epidemiology.

[bib54] Rubin S.S. (1993). Handbook of bereavement: Theory, research, and intervention.

[bib55] Rubin S.S. (1999). The two-track model of bereavement: Overview, retrospect, and prospect. Death Studies.

[bib56] Schiffman D.D. (2019).

[bib57] Shear M.K. (2015). Complicated grief. New England Journal of Medicine.

[bib58] Song J., Mailick M.R., Greenberg J.S., Floyd F.J. (2019). Mortality in parents after the death of a child. Social Science & Medicine.

[bib59] Stroebe M., Schut H., Stroebe W. (2007). Health outcomes of bereavement. The Lancet.

[bib60] Temes R. (1992).

[bib61] Van den Berg G.J., Lundborg P., Vikström J. (2017). The economics of grief. The Economic Journal.

[bib62] van der Houwen K., Stroebe M., Stroebe W., Schut H., Bout J. van den, Meij L.W.D. (2010). Risk factors for bereavement outcome: A multivariate approach. Death Studies.

[bib63] Vance J.C., Najman J.M., Thearle M.J., Embelton G., Foster W., Boyle F.M. (1995). Psychological changes in parents eight months after the loss of an infant from stillbirth, neonatal death, or sudden infant death syndrome—a longitudinal study. Pediatrics.

[bib64] Von Elm E., Altman D.G., Egger M., Pocock S.J., Gøtzsche P.C., Vandenbroucke J.P. (2007). The strengthening the reporting of observational studies in epidemiology (STROBE) statement: Guidelines for reporting observational studies. Bulletin of the World Health Organization.

[bib65] Wall‐Wieler E., Roos L.L., Bolton J. (2018). Duration of maternal mental health‐related outcomes after an infant's death: A retrospective matched cohort study using linkable administrative data. Depression and Anxiety.

[bib66] Walsh F., McGoldrick M. (2013). Bereavement: A family life cycle perspective. Family Science.

[bib67] WHO (2023).

[bib68] Worden J.W. (2018).

[bib69] Zetumer S., Young I., Shear M.K., Skritskaya N., Lebowitz B., Simon N., Reynolds C., Mauro C., Zisook S. (2015). The impact of losing a child on the clinical presentation of complicated grief. Journal of Affective Disorders.

