# Supplementary material for: Bereavement due to child loss, divorce, and depressive mood in older age across European welfare regimes
Source: SSM Popul Health. 2024 Oct 24;28:101721. doi: 10.1016/j.ssmph.2024.101721 (PMC11582450; doi:10.1016/j.ssmph.2024.101721)
Supplement: Multimedia component 1 [file mmc1.docx]

**Supplementary Information (Online)**

Bereavement due to child loss, divorce, and depressive mood in older age

across European welfare regimes

**The Survey of Health, Aging, and Retirement in Europe (SHARE)**

The Survey of Health, Aging, and Retirement in Europe (SHARE) (Börsch-Supan et al., 2013) is a household-based prospective multi-population (cross-national, based on probability samples, and nationally representative) panel database of microdata (multidisciplinary and comparable over time). The target population is represented by the community-dwelling European population in the transition phase (aged 50-60) or in the older age (60+). After registration and approval, data are available to the scientific community (<http://www.share-project.org>). SHARE started in 2004; since then, participants have been interviewed every 2 years, for a total of 8 waves (2004/05; 2006/07; 2008/09; 2010/11; 2013/14; 2015/16; 2017/18; 2019/20). The interview investigates participants’ health, social and economic condition, and is repeated longitudinally to observe the characteristics of the aging process. All questionnaires are harmonized cross-nationally.


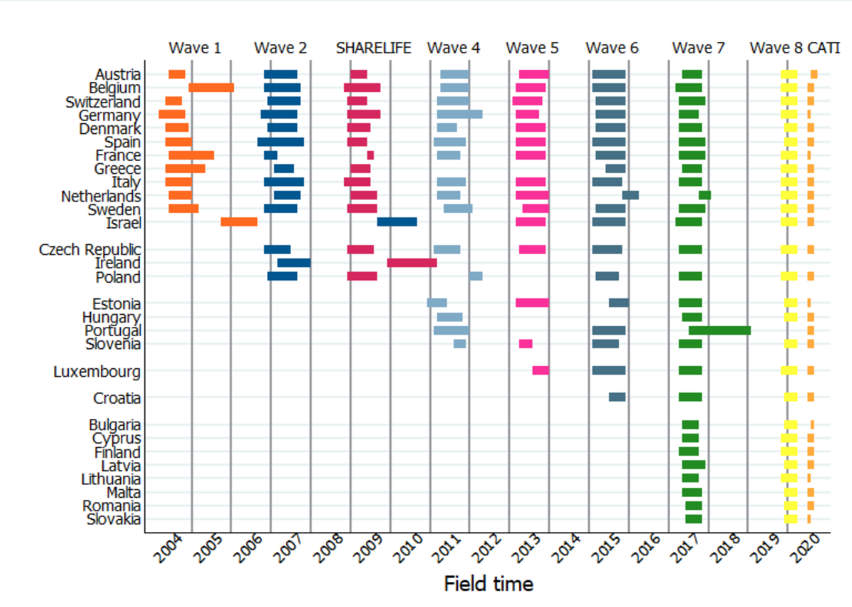


**Waves overview**. From <https://share-eric.eu/data/data-documentation/waves-overview>

The inclusion criteria to participate in SHARE are a minimum of 50 years of age, and not having been incarcerated, hospitalized, or out of the country during the entire survey period, not being unable to speak the country’s language(s) or have moved to an unknown address. Current partners living in the household are also interviewed, regardless of their age. The longitudinal sample is made up of all SHARE respondents who were part of any previous wave. Refreshment samples are drawn to increase the net sample size.

Participants interviewed for the first time were born in 1954 or earlier in wave 1 (2004/05), in 1956 or earlier in wave 2 (2006/07), in 1960 or earlier in wave 4 (2010/11), in 1963 or earlier in wave 5 (2013-14), in 1965 or earlier in wave 6 (2015-16), in 1967 or earlier in wave 7 (2017-18), in 1969 or earlier in wave 8. Wave 3 (SHARELIFE) consists of a retrospective assessment of the life histories of the participants interviewed in wave 1 or 2.

**E-Tables**

**E-Table 1.** Participants for each country at baseline (wave 1 or wave 2) in the analytical study sample as a whole and stratified by gender.

| **Geographical area** | **All** (n = 22,959) | **Men** (n = 9,931) | **Women** (n = 13,028) |
| --- | --- | --- | --- |
| Nordic countries |  |  |  |
| *Denmark* | 1,801(7.8%) | 781(7.9%) | 1,020(7.8%) |
| *Sweden* | 1,638(7.1%) | 726(7.3%) | 912(7.0%) |
| Central European countries |  |  |  |
| *Austria* | 790(3.4%) | 308(3.1%) | 482(3.7%) |
| *Belgium* | 2,443(10.6%) | 1,072(10.8%) | 1,371(10.5%) |
| *France* | 2,072(9.0%) | 887(8.9%) | 1,185(9.1%) |
| *Germany* | 1,636(7.1%) | 739(7.4%) | 897(6.9%) |
| *Netherlands* | 1,796(7.8%) | 808(8.1%) | 988(7.6%) |
| *Switzerland* | 1,021(4.5%) | 441(4.4%) | 580(4.5%) |
| Southern European countries |  |  |  |
| *Greece* | 2,590(11.3%) | 1,098(11.1%) | 1,492(11.5%) |
| *Italy* | 2,188(9.5%) | 984(9.9%) | 1,204(9.2%) |
| *Spain* | 1,810(7.9%) | 776(7.8%) | 1,034(7.9%) |
| Eastern European countries |  |  |  |
| *Czech Republic* | 1,602(6.9%) | 649(6.5%) | 953(7.3%) |
| *Poland* | 1,572(6.8%) | 662(6.7%) | 910(6.9%) |

**E-Table 2.** Baseline (wave 1 or wave 2) characteristics of the analytical study sample as a whole and stratified by gender.

**(A) All countries**

| **Characteristic** | **All** (n = 22,959) | **Men** (n = 9,931) | **Women** (n = 13,028) |
| --- | --- | --- | --- |
| Age (years)^1^ | 62(55 – 70) | 63(56 – 70) | 61(55 – 70) |
| Marital status |  |  |  |
| *Living with partner* | 17,873(77.85%) | 8,702(87.62%) | 9,171(70.39%) |
| *Not living with partner* | 5,086(22.15%) | 1,229(12.38%) | 3,857(29.61%) |
| Number of children^1^ | 2(2 – 3) | 2(2 – 3) | 2(2 – 3) |
| Education (years)^1^ | 11 (7 – 13) | 11 (7 – 14) | 10 (6 – 13) |
| Current job situation |  |  |  |
| *Retired* | 10, 740 (46.8%) | 5,548(55.8%) | 5,192(39.8%) |
| *Employed or self-employed* | 7, 081 (30.8%) | 3,624(36.5%) | 3,457(26.5%) |
| *Unemployed* | 686 (3.0%) | 308(3.1%) | 378(2.9%) |
| *Permanently sick* | 772 (3.4%) | 352(3.5%) | 420(3.2%) |
| *Homemaker* | 3,500 (15.2%) | 35(0.3%) | 3,456(26.6%) |
| *Other* | 180 (0.8%) | 64(0.6%) | 116(0.9%) |
| Number of chronic diseases |  |  |  |
| *0* | 6,016(26.2%) | 2,787(28.1%) | 3,229(24.8%) |
| *1* | 7,247(31.6%) | 3,295(33.2%) | 3,952(30.3%) |
| *2+* | 9,696(42.2%) | 3,849(38.8%) | 5,847(44.9%) |
| BMI^1^ | 26.12(23.76 – 29.00) | 26.43(24.46 – 28.73) | 25.78(23.18 – 29.05) |
| Ever smoked daily |  |  |  |
| *No* | 12,163(52.9%) | 3,597(36.2%) | 8,566(65.7%) |
| *Yes* | 10,796(47.0%) | 6,334(63.8%) | 4,462(34.3%) |
| Drinking more than 2 glasses of alcohol almost everyday |  |  |  |
| *No* | 19,309(84.1%) | 7,376(74.3%) | 11,933(91.6%) |
| *Yes* | 3,650(15.9%) | 2,555(25.7%) | 1,095(8.4%) |
| Depressive mood^2^ |  |  |  |
| No | 17,228(75.0%) | 8,388(84.5%) | 8,840(67.8%) |
| Yes | 5,731(25.0%) | 1,543(15.5%) | 4,188(32.1%) |

^1^ Median(IQR)

^2^ Euro-D scale score more or equal to 4 (cut-off)

**(B) Central European countries**

| **Characteristic** | **All** (n = 9,758) | **Men** (n = 4,255) | **Women** (n = 5,503) |
| --- | --- | --- | --- |
| Age (years)^1^ | 62(55 – 70) | 62 (65 – 70) | 61 (55 – 70) |
| Marital status |  |  |  |
| *Living with partner* | 7,436(76.2%) | 3,659(86.0%) | 3,777(68.6%) |
| *Not living with partner* | 2,322(23.8%) | 596(14.0%) | 1,726(31.4%) |
| Number of children^1^ | 2(2 – 3) | 2 (2 – 3) | 2 (2 – 3) |
| Education (years)^1^ | 12 (8 – 13) | 9 (12 – 14) | 11 (8 – 13) |
| Current job situation |  |  |  |
| *Retired* | 4,377(44.9%) | 2,336(54.9%) | 2,041(37.1%) |
| *Employed or self-employed* | 3,135(32.1%) | 1,564(36.8%) | 1,571(28.5%) |
| *Unemployed* | 334(3.4%) | 147(3.5%) | 187(3.4%) |
| *Permanently sick* | 319(3.3%) | 155(3.6%) | 164(3.0%) |
| *Homemaker* | 1,525(15.6%) | 27(0.6%) | 1,498(27.2%) |
| *Other* | 68(0.7%) | 26(0.6%) | 42(0.8%) |
| Number of chronic diseases |  |  |  |
| *0* | 2,798(28.7%) | 1,269(29.8%) | 1,529(27.8%) |
| *1* | 3,226(33.1%) | 1,443(33.9%) | 1,783(32.4%) |
| *2+* | 3,734(38.3%) | 1,543(36.3%) | 2,191(39.8%) |
| BMI^1^ | 25.76(23.44 – 28.68) | 26.23(24.22 – 28.78) | 25.34(22.67 – 28.62) |
| Ever smoked daily |  |  |  |
| *No* | 5,175(53.0%) | 1,529(35.9%) | 3,646(66.5%) |
| *Yes* | 4,583(47.0%) | 2,726(64.1%) | 1,857(33.8%) |
| Drinking more than 2 glasses of alcohol almost everyday |  |  |  |
| *No* | 8,156(83.6%) | 3,127(73.5%) | 5,029(91.4%) |
| *Yes* | 1,602(16.4%) | 1,128(26.5%) | 474(8.6%) |
| Depressive mood^2^ |  |  |  |
| No | 7,566(77.5%) | 3,660(86.0%) | 3,906(71.0%) |
| Yes | 2,192(22.5%) | 595(14.0%) | 1,597(29.0%) |

^1^ Median(IQR)

^2^ Euro-D scale score more or equal to 4 (cut-off)

**(C) Nordic European countries**

| **Characteristic** | **All** (n = 3,439) | **Men** (n = 1,507) | **Women** (n = 1,932) |
| --- | --- | --- | --- |
| Age (years)^1^ | 62 (55 – 70) | 62 (56 – 70) | 61 (55 – 70) |
| Marital status |  |  |  |
| *Living with partner* | 2,632(76.5%) | 1,266(84.0%) | 1,366(70.7%) |
| *Not living with partner* | 807(23.5%) | 241(16.0%) | 566(29.3%) |
| Number of children^1^ | 2(2 – 3) | 2(2 – 3) | 2(2 – 3) |
| Education (years)^1^ | 12(9 - 15 | 12(9 – 15) | 12(8 – 15) |
| Current job situation |  |  |  |
| *Retired* | 1,619(47.1%) | 705(46.8%) | 914(47.3%)) |
| *Employed or self-employed* | 1,562(45.4%) | 721(47.8%) | 841(43.5%) |
| *Unemployed* | 96(2.8%) | 41(2.7%) | 55(2.8%) |
| *Permanently sick* | 113(3.3%) | 37(2.5%) | 76(3.9%) |
| *Homemaker* | 40(1.2%) | 0(0.00%) | 40(2.1%) |
| *Other* | 9(0.3%) | 3(0.2%) | 6(0.3%) |
| Number of chronic diseases |  |  |  |
| *0* | 944(27.5%) | 454(30.1%) | 490(25.4%) |
| *1* | 1,128(32.8%) | 509(33.8%) | 619(32.0%) |
| *2+* | 1,367(39.7%) | 544(36.1%) | 823(42.6%) |
| BMI^1^ | 25.31(23.12 – 27.99) | 25.79(23.94 – 28.07) | 24.84(22.49 – 27.85) |
| Ever smoked daily |  |  |  |
| *No* | 1,449(42.1%) | 568(37.7%) | 881(45.6%) |
| *Yes* | 1,990(57.8%) | 939(62.3%) | 1,051(54.4%) |
| Drinking more than 2 glasses of alcohol almost everyday |  |  |  |
| *No* | 2,984(86.8%) | 1,246(82.7%) | 1,738(90.0%) |
| *Yes* | 455(13.2%) | 261(17.3%) | 194(10.0%) |
| Depressive mood^2^ |  |  |  |
| No | 2,897(84.2%) | 1,363(90.4%) | 1,534(79.4%) |
| Yes | 542(15.8%) | 144(9.6%) | 398(20.6%) |

^1^ Median(IQR)

^2^ Euro-D scale score more or equal to 4 (cut-off)

**(D) Southern European countries**

| **Characteristic** | **All** (n = 6,588) | **Men** (n = 2,858) | **Women** (n = 3,730) |
| --- | --- | --- | --- |
| Age (years)^1^ | 62(55 – 71) | 64(57 – 71) | 61(54 – 70) |
| Marital status |  |  |  |
| *Living with partner* | 5,461(82.9%) | 2,646(92.6%) | 2,815(75.5%) |
| *Not living with partner* | 1,127(17.1%) | 212(7.4%) | 915(24.5%) |
| Number of children^1^ | 2(2 – 3) | 2(2 – 3) | 2(2 – 3) |
| Education (years)^1^ | 6(5 – 12) | 8(5 – 12) | 6(5 – 11) |
| Current job situation |  |  |  |
| *Retired* | 2,772(42.1%) | 1,740(60.9%) | 1,032(27.7%) |
| *Employed or self-employed* | 1,617(24.5%) | 958(33.5%) | 659(17.7%) |
| *Unemployed* | 159(2.4%) | 81(2.8%) | 78(2.1%) |
| *Permanently sick* | 129(2.0%) | 55(1.9%) | 74(2.0%) |
| *Homemaker* | 1,861(28.2%) | 7(0.2%) | 1,854(49.7%) |
| *Other* | 50(0.8%) | 17(0.6%) | 33(0.9%) |
| Number of chronic diseases |  |  |  |
| *0* | 1,657(25.1%) | 801(28.0%) | 856(22.9%) |
| *1* | 2,030(30.8%) | 946(33.1%) | 1,084(29.1%) |
| *2+* | 2,901(44.0%) | 1,111(38.9%) | 1,790(48.0%) |
| BMI^1^ | 26.57(24.31 – 29.38) | 26.77(24.91 – 29.29) | 26.38(23.79 – 29.38) |
| Ever smoked daily |  |  |  |
| *No* | 3,831(58.1%) | 1,033(36.1%) | 2,798(75.0%) |
| *Yes* | 2,757(42.0%) | 1,825(64.0%) | 932(25.0%) |
| Drinking more than 2 glasses of alcohol almost everyday |  |  |  |
| *No* | 5,593(84.9%) | 2,108(73.8%) | 3,485(93.4%) |
| *Yes* | 995(15.1%) | 750(26.2%) | 245(6.6%) |
| Depressive mood^2^ |  |  |  |
| No | 4,665(70.8%) | 2,363(82.7%) | 2,302(61.7%) |
| Yes | 1,923(29.2%) | 495(17.3%) | 1,428(38.3%) |

^1^ Median(IQR)

^2^ Euro-D scale score more or equal to 4 (cut-off)

**(E) Eastern European countries**

| **Characteristic** | **All** (n = 3,174) | **Men** (n = 1,311) | **Women** (n = 1,863) |
| --- | --- | --- | --- |
| Age (years)^1^ | 62(56 – 70) | 63(57 – 70) | 61(56 – 70) |
| Marital status |  |  |  |
| *Living with partner* | 2,344(73.8%) | 1,131(82.3%) | 1,213(65.1%) |
| *Not living with partner* | 830(26.1%) | 180(13.7%) | 650(34.9%) |
| Number of children^1^ | 2(2 – 3) | 2(2 – 3) | 2(2 – 3) |
| Education (years)^1^ | 11(8 – 12) | 11(9 – 13) | 11(8 – 12) |
| Current job situation |  |  |  |
| *Retired* | 1,972(62.1%) | 767(58.5%) | 1,205(64.5%) |
| *Employed or self-employed* | 767(24.2%) | 381(29.1%) | 386(20.7%) |
| *Unemployed* | 97(3.1%) | 39(3.0%) | 58(3.1%) |
| *Permanently sick* | 211(6.6%) | 105(8.0%) | 106(5.7%) |
| *Homemaker* | 74(2.3%) | 1(0.1%) | 73(3.9%) |
| *Other* | 53(1.7%) | 18(1.4%) | 35(1.9%) |
| Number of chronic diseases |  |  |  |
| *0* | 617(19.4%) | 263(20.1%) | 354(19.0%) |
| *1* | 863(27.2%) | 397(30.3%) | 466(25.0%) |
| *2+* | 1,694(53.4%) | 651(49.7%) | 1,043(56.0%) |
| BMI^1^ | 27.04(24.57 – 30.08) | 26.85(24.69 – 29.41) | 27.31(24.34 – 30.47) |
| Ever smoked daily |  |  |  |
| *No* | 3,831(58.1%) | 467(35.6%) | 1,241(66.6%) |
| *Yes* | 2,757(41.8%) | 844(64.4%) | 622(33.4%) |
| Drinking more than 2 glasses of alcohol almost everyday |  |  |  |
| *No* | 2,576(81.2%) | 895(68.3%) | 1,681(90.2%) |
| *Yes* | 598(18.8%) | 416(31.7%) | 182(9.8%) |
| Depressive mood^2^ |  |  |  |
| No | 2,100(66.2%) | 1,002(76.4%) | 1,098(59.0%) |
| Yes | 1,074(33.8%) | 309(23.6%) | 765(41.1%) |

^1^ Median(IQR)

^2^ Euro-D scale score more or equal to 4 (cut-off)

**E-Table 3.** Estimates of effects of exposure to (i) child loss according to age; (ii) time since child death; (iii) time since divorce, and association with presence of depressive symptoms at transition/older age (square-root transformed EURO-D scores).

| **Unadjusted** |  |  | **Exposure*welfare regime** | | | |
| --- | --- | --- | --- | --- | --- | --- |
| **Exposure** | **Level** | **All countries** | **Central European welfare** | **Nordic European welfare** | **Southern European welfare** | **Eastern European welfare** |
| Child loss according to age | No child loss | Ref. |  |  |  | R |
|  | Child loss, decease occurred at age 0-6 | 0.31[0.22, 0.41]* | 0.07[-0.06, 0.20] | -0.07[-0.20, 0.06] | 0.19[-0.01, 0.39] | 0.22[0.008, 0.44]* |
|  | Child loss, decease occurred at age 7-17 | 0.14[0.05, 0.23]* | 0.08[-0.06, 0.23] | -0.08[-0.23, 0.06] | -0.03[-0.22, 0.17] | 0.18[0.09, 0.26]* |
|  | Child loss, decease occurred at age 18+ | 0.33[0.22, 0.44]* | -0.07[-0.15, 0.004] | 0.07[-0.004, 0.15] | 0.37[0.15, 0.59]* | 0.15[0.02, 0.28]* |
| Time since child death | No child loss | Ref. |  |  |  |  |
|  | Child loss, decease occurred less than 6 years from SHARELIFE | 0.60[0.36, 0.85]* | 0.20[-0.24, 0.65] | -0.20[-0.64, 0.24] | 0.28[-0.12, 0.68] | -0.14[-0.60, 0.33] |
|  | Child loss, decease occurred 6-10 years from SHARELIFE | 0.37[0.17, 0.56]* | -0.16[-0.55, 0.23] | 0.16[-0.23, 0.55] | 0.19[-0.19, 0.58] | 0.71[0.35, 1.06]* |
|  | Child loss, decease occurred 10+ years from SHARELIFE | 0.29[0.19, 0.39]* | 0.15[0.07, 0.24]* | 0.34[0.09, 0.62]* | 0.43[0.36, 0.51]* | 0.63[0.49, 0.76]* |
| Time since divorce | No child loss or no divorce | Ref. |  |  |  |  |
|  | Divorce occurred before child loss | 0.33[0.10, 0.55]* | 0.18[-0.21, 0.58] | -0.18[-0.58, 0.21] | 0.50[-0.36, 1.36] | 0.20[-0.20, 0.60] |
|  | Divorce occurred after child loss | 0.26[0.10, 0.42]* | 0.09[-0.11, 0.28] | -0.09[-0.28, 0.11] | 0.03[-0.14, 0.19] | -0.06[-0.72, 0.61] |
|  |  |  |  |  |  |  |
| **Adjusted** |  |  | **Exposure*welfare regime** | | | |
| **Exposure** | **Level** | **All countries** | **Central European welfare** | **Nordic European welfare** | **Southern European welfare** | **Eastern European welfare** |
| Child loss according to age | No child loss | Ref. |  |  |  |  |
|  | Child loss, decease occurred at age 0-6 | 0.26[0.14, 0.38]* | -0.13[-0.47, 0.21] | 0.13[-0.21, 0.48] | 0.07[-0.13, 0.27] | 0.01[-0.19, 0.21] |
|  | Child loss, decease occurred at age 7-17 | 0.06[-0.18, 0.29] | 0.22[-0.26, 0.71] | -0.22[-0.71, 0.26] | -0.33[-0.78, 0.12] | 0.17[-0.08, 0.43] |
|  | Child loss, decease occurred at age 18+ | 0.29[0.23, 0.37]* | -0.06[-0.17, 0.05] | 0.06[-0.05, 0.17] | 0.25[-0.03, 0.54] | 0.04[-0.16, 0.23] |
| Time since child death | No child loss | Ref. |  |  |  |  |
|  | Child loss, decease occurred less than 6 years from SHARELIFE | 0.30[-0.27, 0.87] | 0.65[-0.23, 1.54] | -0.65[-1.54, 0.23] | 0.73[0.17, 1.28]* | -0.65[-1.54, 0.23] |
|  | Child loss, decease occurred 6-10 years from SHARELIFE | 0.35[0.14, 0.55]* | -0.22[-0.53, 0.09] | 0.22[-0.09, 0.53] | 0.10[-0.32, 0.52] | 0.30[-0.18, 0.79] |
|  | Child loss, decease occurred 10+ years from SHARELIFE | 0.27[0.16, 0.39]* | -0.08[-0.41, 0.24] | 0.08[-0.24, 0.41] | 0.08[-0.12, 0.29] | 0.09[-0.22, 0.41] |
| Time since divorce | No child loss or no divorce | Ref. |  |  |  |  |
|  | Divorce occurred before child loss | 0.41[0.22, 0.60]* | -0.23[-0.49, 0.04] | 0.23[-0.04, 0.49] | -0.83[-0.99, -0.67]* | -0.19[-0.45, 0.07] |
|  | Divorce occurred after child loss | 0.37[0.02, 0.73]* | -0.01[-0.56, 0.53] | 0.01[-0.53, 0.55] | NA | -0.29[-1.06, 0.47] |

*OLS Regression with robust estimation of the standard error. Adjusted by gender, age, educational level, and birth cohort. Observations were clustered by country for robust standard error estimation.*

**E-Table 4.** Estimates of effects of exposure to child loss combined with divorce occurrence and association with presence of depressive symptoms at transition/older age (square-root transformed EURO-D scores) stratified by gender.

| **Unadjusted** |  |  |  |  |  |  |
| --- | --- | --- | --- | --- | --- | --- |
| **Females** |  |  | **Exposure*welfare regime** | | | |
| **Exposure** | **Level** | **All countries** | **Central European welfare** | **Nordic European welfare** | **Southern European welfare** | **Eastern European welfare** |
| Child loss combined with divorce occurrence | No child loss, no divorce | Ref. |  |  |  |  |
|  | Child loss, no divorce | 0.31[0.21, 0.41]* | 0.17[0.07, 0.27]* | -0.17[-0.27, -0.07]* | 0.58[0.45, 0.70] | 0.55[0.31, 0.80]* |
|  | No child loss, divorce | 0.02[-0.08, 0.12] | 0.19[0.06, 0.32]* | -0.19[-0.32, -0.06]* | 0.15[-0.002, 0.29] | 0.10[-0.26, 0.47] |
|  | Child loss, divorce | 0.31[0.19, 0.43]* | 0.18[-0.05, 0.43] | -0.18[-0.43, 0.05] | 0.29[-0.18, 0.76] | 0.57[0.16, 0.98]* |
| **Males** |  |  |  |  |  |  |
| Child loss combined with divorce occurrence | No child loss, no divorce | Ref. |  |  |  |  |
|  | Child loss, no divorce | 0.24[0.14, 0.35]* | 0.13[-0.09, 0.36] | -0.13[-0.36, 0.09] | 0.34[0.10, 0.58]* | 0.61[0.16, 1.05]* |
|  | No child loss, divorce | 0.03[-0.04, 0.11] | 0.23[0.12, 0.33]* | -0.22[-0.33, -0.11]* | 0.19[0.007, 0.37]* | 0.34[-0.23, 0.92] |
|  | Child loss, divorce | 0.14[-0.09, 0.38]* | 0.60[0.29, 0.92]* | -0.60[-0.92, -0.29]* | 0.51[0.16, 0.85]* | 0.66[0.25, 1.07]* |
|  |  |  |  |  |  |  |
| **Adjusted** |  |  |  |  |  |  |
| **Females** |  |  | **Exposure*welfare regime** | | | |
| **Exposure** | **Level** | **All countries** | **Central European welfare** | **Nordic European welfare** | **Southern European welfare** | **Eastern European welfare** |
| Child loss combined with divorce occurrence | No child loss, no divorce | Ref. |  |  |  |  |
|  | Child loss, no divorce | 0.11[0.01, 0.22]* | -0.016[-0.25, 0.21] | -0.06[-0.28, 0.17] | 0.14[0.007, 0.27]* | 0.02[-0.16, 0.19] |
|  | No child loss, divorce | 0.005[-0.09, 0.10] | 0.04[-0.12, 0.21] | -0.07[-0.24, 0.09] | 0.30[0.12, 0.48]* | -0.07[-0.24, 0.11] |
|  | Child loss, divorce | 0.35[0.19, 0.49]* | -0.04[-0.33, 0.25] | 0.02[-0.27, 0.32] | -1.16[-1.39, -0.92]* | -0.20[-0.42, 0.01] |
| **Males** |  |  |  |  |  |  |
| Child loss combined with divorce occurrence | No child loss, no divorce | Ref. |  |  |  |  |
|  | Child loss, no divorce | 0.22[0.08, 0.35]* | -0.12[-0.39, 0.15] | 0.12[-0.15, 0.39] | 0.10[-0.12, 0.32] | 0.14[-0.03, 0.32] |
|  | No child loss, divorce | 0.05[-0.03, 0.13] | 0.01[-0.09, 0.12] | -0.01[-0.12, 0.09] | 0.26[0.06, 0.46]* | -0.05[-0.20, 0.10] |
|  | Child loss, divorce | 0.36[0.07, 0.64]* | 0.23[-0.91, 0.38] | -0.23[-1.38, 0.91] | NA | 0.02[-1.08, 1.12] |

*OLS Regression with robust estimation of the standard error stratified by gender. Adjusted by age, birth cohort, gender, education, marital status, smoking, drinking, chronic diseases. Observations were clustered by country for robust standard error estimation.*

**E-Table 5.** Estimates of effects of exposure to child loss combined with divorce occurrence and association with presence of depressive symptoms (square-root transformed EURO-D scores) in the transition phase or older age.

| **Unadjusted** |  |  |  |  |  |  |  |
| --- | --- | --- | --- | --- | --- | --- | --- |
| **Transition phase (aged 50-60 years)** |  |  | **Exposure*welfare regime** | | | | |
| **Exposure** | **Level** | **All countries** | **Central European welfare** | **Nordic European welfare** | **Southern European welfare** | **Eastern European welfare** |  |
| Child loss combined with divorce occurrence | No child loss, no divorce | Ref. |  |  |  |  |  |
|  | Child loss, no divorce | 0.26[0.13, 0.38]* | 0.003[-0.16, 0.17] | -0.003[-0.17, 0.17] | 0.44[0.17, 0.70]* | 0.31[0.07, 0.54]* |  |
|  | No child loss, divorce | 0.12[0.05, 0.19]* | 0.05[-0.03, 0.14] | -0.05[-0.14, 0.03] | 0.007[-0.07, 0.08] | -0.11[-0.18, -0.04]* |  |
|  | Child loss, divorce | 0.63[0.43, 0.83]* | 0.04[-0.27, 0.36] | -0.04[-0.36, 0.27] | -0.43[-0.70, -0.16]* | -0.28[-0.79, 0.22] |  |
|  |  |  |  |  |  |  |  |
| **Older age (aged 60+)** |  |  |  |  |  |  |  |
| Child loss combined with divorce occurrence | No child loss, no divorce | Ref. |  |  |  |  |  |
|  | Child loss, no divorce | 0.29[0.21, 0.38]* | -0.02[-0.18, 0.13] | 0.02[-0.13, 0.18] | 0.06[-0.14, 0.26] | 0.07[-0.15, 0.28] |  |
|  | No child loss, divorce | -0.07[-0.19, 0.04] | 0.12[0.01, 0.23]* | -0.12[-0.22, -0.01]* | -0.13[-0.19, -0.06]* | -0.21[-0.31, -0.09] |  |
|  | Child loss, divorce | 0.14[-0.04, 0.32] | 0.25[0.04, 0.45]* | -0.25[-0.46, 0.05]* | 0.29[0.06, 0.51]* | 0.26[0.05, 0.48]* |  |
|  |  |  |  |  |  |  |  |
| **Adjusted** |  |  |  |  |  |  |  |
| **Transition phase (aged 50-60 years)** |  |  | **Exposure*welfare regime** | | | | |
| **Exposure** | **Level** | **All countries** | **Central European welfare** | **Nordic European welfare** | **Southern European welfare** | **Eastern European welfare** |  |
| Child loss combined with divorce occurrence | No child loss, no divorce | Ref. |  |  |  |  |  |
|  | Child loss, no divorce | 0.30[0.19, 0.41]* | 0.03[-0.42, 0.47] | 0.08[-0.36, 0.51] | 0.12[-0.38, 0.64] | 0.13[-0.26, 0.51] |  |
|  | No child loss, divorce | -0.07[-0.22, 0.08] | -0.07[-0.22, 0.08] | 0.07[-0.08, 0.22] | 0.17[-0.17, 0.52] | -0.15[-0.29, -0.01]* |  |
|  | Child loss, divorce | 0.44[0.11, 0.77]* | 0.47[-0.11, 1.04] | -0.44[-0.99, 0.11] | NA | -0.18[-0.87, 0.49] |  |
| **Older age (aged 60+)** |  |  |  |  |  |  |  |
| Child loss combined with divorce occurrence | No child loss, no divorce | Ref. |  |  |  |  |  |
|  | Child loss, no divorce | 0.18[0.09, 0.27]* | -0.06[-0.29, 0.17] | 0.06[-0.17, 0.29] | 0.13[-0.01, 0.27] | 0.09[-0.06, 0.25] |  |
|  | No child loss, divorce | -0.04[-0.16, 0.07] | 0.16[0.001, 0.33]* | -0.16[-0.33, -0.001]* | 0.46[0.36, 0.57]* | -0.15[-0.25, -0.05]* |  |
|  | Child loss, divorce | 0.27[0.12, 0.42]* | -0.22[-0.53, 0.08] | 0.22[-0.08, 0.53] | -0.79[-1.15, -0.44]* | -0.19[-0.55, 0.15] |  |

*OLS Regression with robust estimation of the standard error stratified by age class. Adjusted by age, birth cohort, gender, education, marital status, smoking, drinking, chronic diseases. Observations were clustered by country for robust standard error estimation.*

**E-Table 6.** Sensitivity analysis. Logistic Regression with robust estimation of the standard error. Estimates of effects of exposure to child loss combined with divorce occurrence, and association with presence of depressive symptoms in the older age (EURO-D dichotomized scores).

| **Unadjusted** |  |  | **Exposure*Welfare** | | | | |
| --- | --- | --- | --- | --- | --- | --- | --- |
| **Exposure** | **Level** | **All countries** | **Central European welfare** | **Nordic European welfare** | **Southern European welfare** | **Eastern European welfare** |  |
| Child loss combined with divorce occurrence | No child loss, no divorce | Ref. |  |  |  |  |  |
|  | Child loss, no divorce | 1.33[1.07, 1.65]* | 0.73[0.63, 0.86]* | 0.98[0.85, 1.15] | 1.05[0.83, 1.28] | 1.31[0.90, 1.89] |  |
|  | No child loss, divorce | 0.73[0.62, 0.87]* | 1.25[1.07, 1.47]* | 1.14[0.93, 1.39] | 0.79[0.71, 0.87]* | 0.88[0.79, 0.97]* |  |
|  | Child loss, divorce | 1.41[1.08, 1.85]* | 1.17[0.86, 1.58] | 0.94[0.72, 1.22] | 1.33[1.02, 1.74]* | 0.68[0.41, 1.15] |  |
| **Adjusted** |  |  | **Exposure*Welfare** | | | | |
| **Exposure** | **Level** | **All countries** | **Central European welfare** | **Nordic European welfare** | **Southern European welfare** | **Eastern European welfare** |  |
| Child loss combined with divorce occurrence | No child loss, no divorce | Ref. |  |  |  |  |  |
|  | Child loss, no divorce | 1.43[0.93, 1.41]* | 0.37[0.30, 0.46]* | 0.35[0.27, 0.46]* | 9.65[6.64, 14.02]* | 0.67[0.53, 1.15] |  |
|  | No child loss, divorce | 0.75[0.62, 0.92]* | 0.46[0.33, 0.62]* | 0.49[0.32, 0.75]* | 12.37[6.24, 24.53]* | 0.36[0.27, 0.47]* |  |
|  | Child loss, divorce | 1.51[1.00, 2.26]* | 13.88[8.13, 23.7]* | 11.81[5.25,26.59]* | 0.001[0.0003,0.005]* | 5.20[2.60, 10.39]* |  |

*Adjusted by age, gender, birth cohort, education, marital status, smoking, drinking, chronic diseases. Observations were clustered by country for robust standard error estimation.*

*Odds ratios with confidence intervals are reported in the table.*

**E-Table 7.** Sensitivity analysis. Logistic Regression with robust estimation of the standard error. Estimates of effects of exposure to child loss without considering divorce occurrence, and association with presence of depressive symptoms in the older age (EURO-D dichotomized scores).

| **Unadjusted** |  |  | **Exposure*Welfare** | | | | |
| --- | --- | --- | --- | --- | --- | --- | --- |
| **Exposure** | **Level** | **All countries** | **Central European welfare** | **Nordic European welfare** | **Southern European welfare** | **Eastern European welfare** |  |
| Child loss | No child loss | Ref. |  |  |  |  |  |
|  | Child loss | 0.37[0.28, 0.47] | -0.14[-0.38, 0.10] | 0.14[-0.10, 0.38] | 0.16[-0.13, 0.46] | 0.16[-0.13, 0.45] |  |
| **Adjusted** |  |  | **Exposure*Welfare** | | | | |
| **Exposure** | **Level** | **All countries** | **Central European welfare** | **Nordic European welfare** | **Southern European welfare** | **Eastern European welfare** |  |
| Child loss | No child loss | Ref. |  |  |  |  |  |
|  | Child loss | 0.23[0.14, 0.31] | -0.01[-0.21, 0.19] | 0.0.1[-0.19, 0.21] | 0.14[-0.12, 0.39] | 0.12[-0.13, 0.37] |  |

*Adjusted by age, gender, birth cohort, education, marital status, smoking, drinking, chronic diseases. Observations were clustered by country for robust standard error estimation.*

*Odds ratios with confidence intervals are reported in the table.*

**E-Figures**

**E-Figure 1.** Illustration of the main features of the design of the study. The exposure (past bereavement due to child loss and divorce from partner) were retrospectively assessed on wave 3 (SHARELIFE). The outcome (EURO-D) scores was assessed in all the other waves.

**
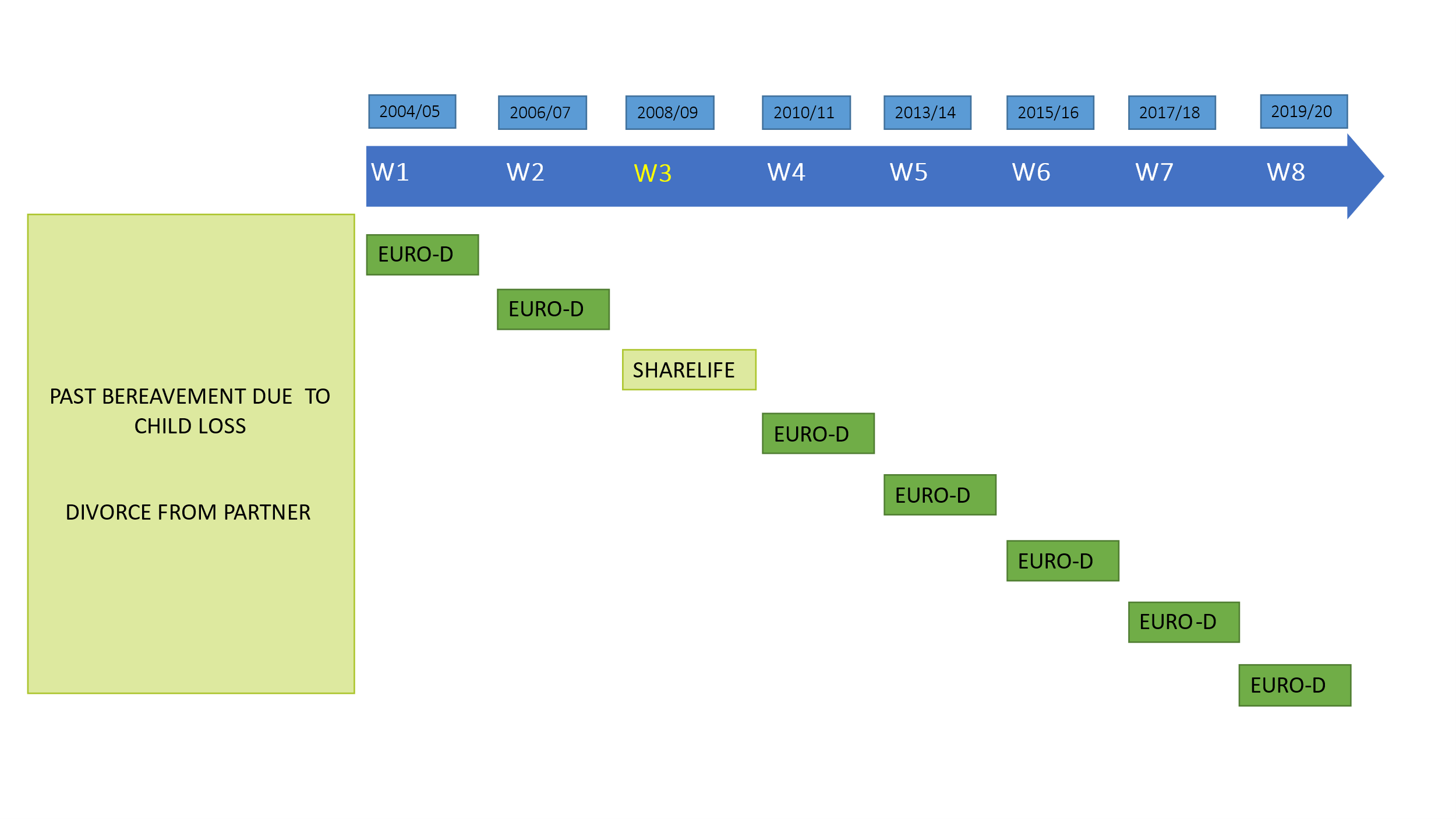
**

**E-Figure 2.** Distribution of EURO-D scores at baseline by welfare regime and gender.

Central European countries


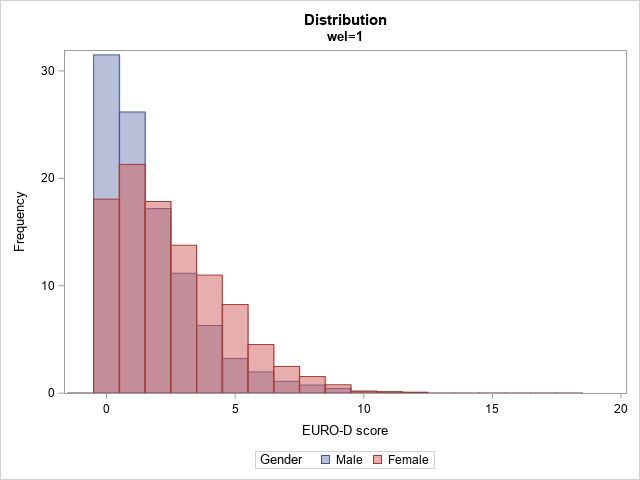


Nordic European countries


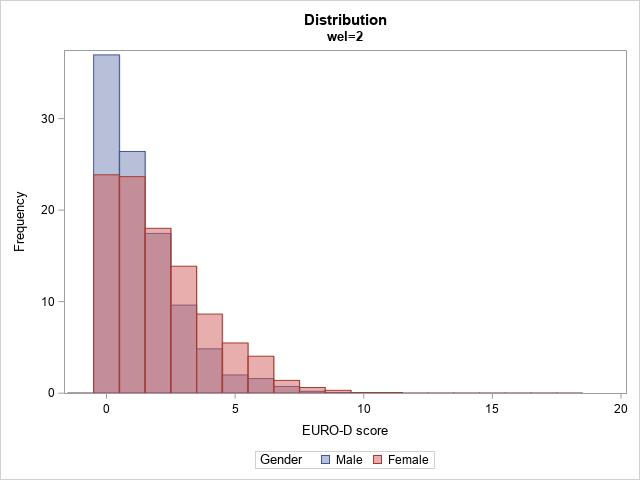


Southern European countries


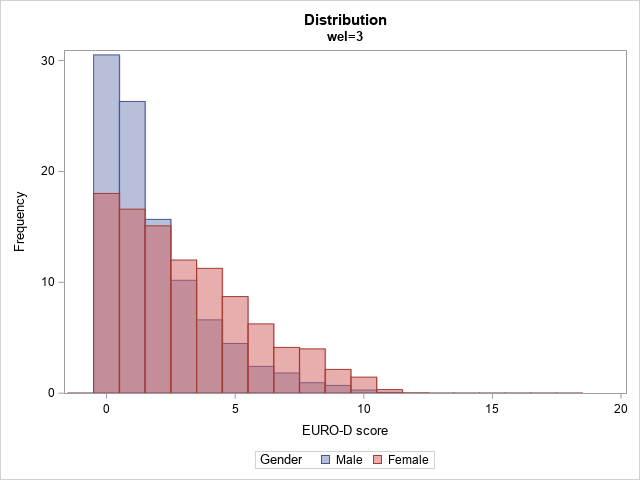


Eastern European countries


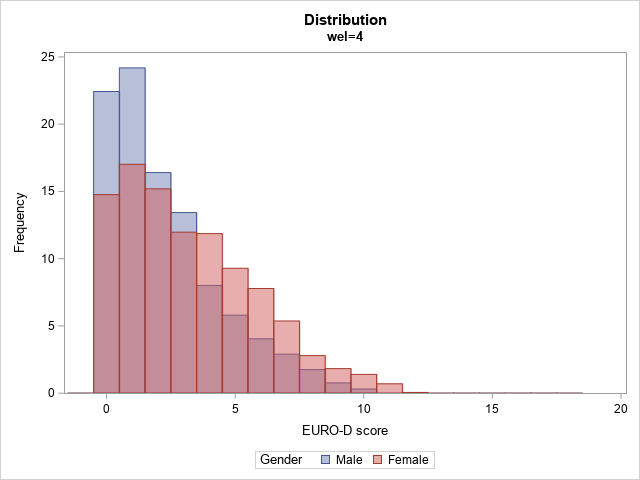


**E-Figure 3.** Beta coefficients (unstandardized and standardized ) from OLS regression of effects of exposure to child loss/divorce in interaction with welfare regime (Exposure x Welfare regime) on presence of depressive symptoms (square-root transformed EURO-D scores) at baseline (wave 1 or 2).

For each graph, estimates have been obtained by switching the reference level relative to welfare regime to *all the other levels* and then averaging.

**E-Figure 4.** Beta coefficients (unstandardized) from GEEs (longitudinal effects) of effects of exposure to child loss/divorce in interaction with age and welfare regime (Exposure x Age x Welfare regime) on presence of depressive symptoms (square-root transformed EURO-D scores) at baseline (wave 1 or 2).

For each graph, estimates have been obtained by switching the reference level relative to welfare regime to *all the other levels* and then averaging.
